# The Teaching of Surgery in America

**Published:** 1901-01-05

**Authors:** 


### THE TEACHING OF SURGERY IN AMERICA.

IN the recently issued Medical and Surgical Reports of the Boston City Hospital there is a paper on the teaching of surgery, which is of some interest as showing how thoroughly this part of the work of a hospital is gone into in America. The principal methods in vogue would seem to be by didactic lectures, clinical lectures, operations in the theatre, sectional teaching in the out-patient department, and consultations sometimes by the students and sometimes by the surgeons. It is made evident that no faith is placed in the idea that surgery can be learned by the old plan of walking the hospitals, or, in other words, by watching the practice of the surgeons. If surgery is to be learnt it must be taught.

Besides the work in the operation theatre, where everything is done to demonstrate to the students the nature of the case which is to be operated on, the main teaching centres in the clinics and the didactic lectures. Perhaps no more important advance has been made in the surgical clinics than in the grouping of the cases to be shown. A careful system of recording interesting cases of a common disease or injury has resulted in enabling the teacher to illustrate his remarks by the exhibition of a large number of cases, in giving a lecture on fracture of the leg for example to show first recent fractures of both bones of the leg, of the fibula, a Pott's fracture, a compound fracture; then a series of cases illustrating fracture of the leg at the end of ten days, at the end of three weeks, six months, and finally one and-a-half or two years. The sequence of presentation of material has been carefully studied, and attention has been paid to the bringing to the student's mind the natural order of injury and disease. On one occasion twenty-eight cases of tracheotomy and intubation were shown to the students, and another time thirty-two cases of appendicitis, which were recent, were shown to the students, while on another occasion fifteen cases of fracture of the skull of various types and conditions, from a simple linear fracture to a compound depressed fracture, were shown.

Another point in the clinical lectures is the use which is made of pathological material, the students being constantly taught to explain clinical phenomena by pathological conditions, and fresh pathological material being shown wherever possible. For this purpose another important step has been the use of clinical material to illustrate corelative didactic lectures. For example, a lecture is given at the medical school on appendicitis on Monday; on Tuesday the students are shown five to fifteen cases illustrating the various stages of the disease; on Wednesday another didactic lecture is given finishing the subject. On Thursday, again, clinical material is shown and operations on appendicitis are done. The following week a recitation on the whole subject is given. This concentration of teaching on a given subject is found to be of the greatest value. Small sections of students, from ten to twenty, are arranged to study cases among the out-patients; but, although valuable in bringing the students into practical touch with the patients, it is considered that such teaching can never supersede the clinical lectures, partly from the fact that the men of most experience cannot afford to devote their entire time to such small classes. In the wards again small sections of fourth year's men are taken, and of course the students, as in England, become dressers, and thus gain personal experience; but the opinion is strongly expressed that it is never desirable to turn students loose into a ward. They must always be under supervision, for although in the abstract it may be true that if a student is thrown upon his own resources he will make more advance than if he is constantly guided, it is neither possible nor desirable to allow students to examine surgical cases, except under the closest supervision.

The authors add, "There are a few students in each class who could be allowed the opportunity of operating under guidance, but they can gain the technique and familiarity with the details by operating upon animals. A careful study of the conditions governing the education of students and the rights of the individual patient convinces me that it is neither desirable nor possible for students to do operations on human beings." It would seem from this article that there is in some American hospitals a greater willingness among the various members of the staff to co-operate with one another, and to make the whole resources of the hospital subservient to the all-important work of teaching, than is commonly found on this side. There is no doubt that much of the "going round the wards" with the surgeons in English hospitals is not only waste of time to the students but very much impedes the surgeons themselves in their scientific work. The treatment of the sick and the instruction of the students are two very different functions, and they cannot generally be both performed at the same time.

